# Diabetic rats with high levels of endogenous dopamine do not show retinal vascular pathology

**DOI:** 10.3389/fnins.2023.1125784

**Published:** 2023-03-22

**Authors:** Rachael S. Allen, Cara T. Khayat, Andrew J. Feola, Alice S. Win, Allison R. Grubman, Kyle C. Chesler, Li He, Jendayi A. Dixon, Timothy S. Kern, P. Michael Iuvone, Peter M. Thule, Machelle T. Pardue

**Affiliations:** ^1^Atlanta VA Center for Visual and Neurocognitive Rehabilitation, Atlanta VA Healthcare System, Decatur, GA, United States; ^2^Department of Biomedical Engineering, Georgia Institute of Technology and Emory University, Atlanta, GA, United States; ^3^Department of Ophthalmology, Emory University, Atlanta, GA, United States; ^4^Department of Pharmacology and Chemical Biology, Emory University, Atlanta, GA, United States; ^5^Department of Pharmacology, Case Western Reserve University, Cleveland, OH, United States; ^6^Veterans Administration Medical Center Research Service, Cleveland, OH, United States; ^7^Gavin Herbert Eye Institute, University of California, Irvine, Irvine, CA, United States; ^8^Section Endocrinology and Metabolism, Atlanta VA Medical Center, Emory University School of Medicine, Decatur, GA, United States

**Keywords:** diabetes, diabetic retinopathy, electroretinogram, Goto-Kakizaki, dopamine, vascular, cognitive

## Abstract

**Purpose:**

Limited research exists on the time course of long-term retinal and cerebral deficits in diabetic rodents. Previously, we examined short term (4–8 weeks) deficits in the Goto-Kakizaki (GK) rat model of Type II diabetes. Here, we investigated the long-term (1–8 months) temporal appearance of functional deficits (retinal, cognitive, and motor), retinal vascular pathology, and retinal dopamine levels in the GK rat.

**Methods:**

In GK rats and Wistar controls, retinal neuronal function (electroretinogram), cognitive function (Y-maze), and motor function (rotarod) were measured at 1, 2, 4, 6, and 8 months of age. In addition, we evaluated retinal vascular function (functional hyperemia) and glucose and insulin tolerance. Retinas from rats euthanized at ≥8 months were assessed for vascular pathology. Dopamine and DOPAC levels were measured *via* HPLC in retinas from rats euthanized at 1, 2, 8, and 12 months.

**Results:**

Goto-Kakizaki rats exhibited significant glucose intolerance beginning at 4 weeks and worsening over time (*p* < 0.001). GK rats also showed significant delays in flicker and oscillatory potential implicit times (*p* < 0.05 to *p* < 0.001) beginning at 1 month. Cognitive deficits were observed beginning at 6 months (*p* < 0.05), but no motor deficits. GK rats showed no deficits in functional hyperemia and no increase in acellular retinal capillaries. Dopamine levels were twice as high in GK vs. Wistar retinas at 1, 2, 8, and 12 months (*p* < 0.001).

**Conclusion:**

As shown previously, retinal deficits were detectable prior to cognitive deficits in GK rats. While retinal neuronal function was compromised, retinal vascular pathology was not observed, even at 12+ months. High endogenous levels of dopamine in the GK rat may be acting as an anti-angiogenic and providing protection against vascular pathology.

## 1. Introduction

Over 537 million people worldwide have diabetes (International Diabetes Federation, 2021), and an estimated 33.9% of adults in the United States have prediabetes (Centers for Disease Control and Prevention, 2015). Diabetes is characterized by hyperglycemia, hyperlipidemia, and inflammation leading to circulatory changes and vascular damage in the kidney, heart, brain, nerves, and retina. Diabetic retinopathy (DR), is one of the most common complications and is the leading cause of blindness in adults 20–74 years of age (Klein, 2007), affecting 22% of people with diabetes (103 million adults) worldwide (Teo et al., 2021). Clinically, DR is diagnosed using vascular pathology, including microaneurysms, cotton-wool spots, intraretinal hemorrhages, and abnormal neovascularization. However, increasing evidence supports the idea that DR affects retinal neurons directly and prior to clinically diagnoseable vascular changes (Barber, 2003; Antonetti et al., 2006; Pardue et al., 2014). Early functional deficits include changes in visual acuity (Aung et al., 2013), contrast sensitivity (Ghirlanda et al., 1997; Aung et al., 2013; Stem et al., 2016), color vision (Ghirlanda et al., 1997; Gella et al., 2015; Wolff et al., 2015), night vision (Ghirlanda et al., 1997; Stem et al., 2016), and electroretinogram latencies (Holopigian et al., 1992, 1997; Shirao and Kawasaki, 1998; Lecleire-Collet et al., 2011; Aung et al., 2013; Pardue et al., 2014; Chesler et al., 2021).

Diabetes causes cognitive and motor deficits in addition to retinal deficits (van Duinkerken et al., 2009; Haan et al., 2012; Sherin et al., 2012; de Almeida Faria et al., 2022a,b; Rhmari Tlemçani et al., 2022). In fact, DR presence and severity have been associated with increased cortical atrophy (Wessels et al., 2006; Hansen et al., 2022), cerebral ischemia (Haan et al., 2012) and microinfarcts (Lee et al., 2019), and structural changes in the brain (Ferguson et al., 2003; Wang et al., 2019). Behavioral changes associated with DR include accelerated cognitive decline (Ding et al., 2010) and deficits in information processing, concentration, and attention (Ferguson et al., 2003; de Almeida Faria et al., 2022a,b; Rhmari Tlemçani et al., 2022). Tracking the temporal progression of deficits allows us to determine whether retinal neuronal changes can be used as an early detector for later cognitive, motor, or retinal vascular changes. While we have shown in an earlier paper that retinal neuronal changes appear prior to cognitive changes in the GK rat in a short-term study (4–8 weeks), a long-term characterization of retinal and cognitive changes (out to 8 months) would be beneficial to the field.

Dopamine deficiency has been identified as a mechanism underlying early retinal function deficits in Type I diabetes (Aung et al., 2014; Pardue and Allen, 2018; Motz et al., 2020). Retinas from diabetic animal models show reductions in dopaminergic amacrine cells (Gastinger et al., 2006) and levels of dopamine and DOPAC (3,4-dihydroxyphenylacetic acid; the metabolite of dopamine) (Aung et al., 2014). Treating with dopamine agonists or L-DOPA (3,4-dihydroxy-L-phenylalanine; the immediate precursor to dopamine) reduces retinal deficits in diabetic rats (Aung et al., 2014; Chesler et al., 2021) and patients with diabetes (Motz et al., 2020). Dopamine disruption has been implicated in damage to other systems in diabetes as well. Diabetes and reduced insulin sensitivity are associated with reduced striatal dopamine (Morris et al., 2011; Caravaggio et al., 2015) and an 80% increased likelihood of developing Parkinson’s disease, a disease characterized by motor deficits due to dopaminergic neuron loss in the basal ganglia (Hu et al., 2007). Depression and cognitive deficits are more common in people with diabetes, possibly because insulin resistance disrupts the dopaminergic systems implicated in both depression and memory (Kleinridders et al., 2015). Finally, giving patients with schizophrenia dopamine antagonists can cause them to develop hyperglycemia and diabetes (Adeneye et al., 2011; Ikegami et al., 2013). A gap in our knowledge is whether dopamine deficiency is involved in early DR in Type II as well as Type I diabetes, and in cognitive and motor deficits as well as retinal deficits. Establishing dopamine disruption as a multi-system consequence of diabetes could reveal new treatment strategies.

A Type II diabetes model was used in this study because 90–95% of patients with diabetes have Type II diabetes (Centers for Disease Control and Prevention, 2021). The Goto-Kakizaki (GK) rat is a polygenic model of Type II diabetes that is neither obese nor insulin dependent (Portha et al., 1991; Ostenson, 2007). This model develops impaired insulin secretion by 2 weeks, impaired fasting hyperglycemia by 4 weeks, and displays retinal (Matsubara et al., 2006; Allen et al., 2019), cognitive (Moreira et al., 2007; Li et al., 2013; Allen et al., 2019), and motor nerve deficits (Suzuki et al., 1990). However, the long-term temporal progression of these deficits has not been characterized. We hypothesized that GK rats would exhibit retinal, cognitive, and motor function deficits, with retinal function deficits appearing first and preceding cognitive and motor deficits and later-stage retinal vascular pathology. Based on previous research by our group and others, we expected GK rats to show: (1) a reduction in retinal dopamine and DOPAC, and (2) retinal vascular pathology that appeared between 6 and 12 months of age. Instead, we found that while retinal deficits in GK rats preceded cognitive deficits and this dysfunction mirrored deficits found in other animal models and in people, GK rats exhibited increased levels of dopamine and DOPAC and did not exhibit the retinal vascular pathology typical of diabetic rats.

## 2. Materials and methods

### 2.1. Animals, diabetes confirmation, and experimental design

Male and female GK (diabetic; *n* = 61) and Wistar (non-diabetic control; *n* = 66) rats were housed in shoe-box style cages on a 12:12 light: dark cycle (light onset at 6:00 AM) with chow and water provided *ad libitum*. Breeders were originally obtained from Charles River, Wilmington, MA, and all rats used in these experiments were from in-house breeding. All procedures were approved by the Atlanta Veterans Affairs Institutional Animal Care and Use Committee and conformed to the *ARVO Statement for the Use of Animals in Ophthalmic and Vision Research* and the *National Institutes of Health Guide for the Care and Use of Laboratory Animals* (National Research Council, 2011) (NIH Publications, 8th edition, updated 2011).

The GK rat is a non-obese, polygenic, spontaneously occurring model of Type II diabetes developed by selectively breeding Wistar rats with the highest blood glucose levels as measured by glucose tolerance test (GTT) (Goto et al., 1976; Ostenson, 2007). As a result, the most dramatic blood glucose differences between wildtype and GK animals are generally observed using GTT (Goto et al., 1976; Ostenson, 2007). GTTs, ITTs (insulin tolerance tests), and body weight were used to confirm hyperglycemia and insulin resistance at 1, 2, and 8 months of age. Insulin supplementation was not needed because GK rats do not lose weight with diabetes, unlike rats with Type I diabetes induced *via* STZ (a toxin that ablates pancreatic beta cells) (Allen et al., 2018).

Retinal neuronal function (electroretinogram, ERG), cognitive function (spontaneous alternation on Y-maze), and motor function (rotarod) were measured at 1, 2, 4, 6, and 8 months of age in GK (*n* = 17) and Wistar (*n* = 23) rats. Functional hyperemia was used to assess retinal vascular function at 8 months. Retinas from rats euthanized at 8, 12, and 12+ months were assessed for vascular pathology or dopamine and DOPAC levels using high performance liquid chromatography (HPLC). Due to limited numbers of rats at 12+ months (*n* = 4–10), the 12 month and 12+ month groups are combined in vascular and dopamine analyses. An additional set of rats that did not receive functional tests was euthanized at 1 month (*n* = 16 for GK and Wistar) and 2 months (*n* = 22 for GK and Wistar) so that HPLC could be performed on tissue from these timepoints.

### 2.2. Glucose and insulin tolerance tests

Glucose tolerance tests and ITTs were used to measure hyperglycemia and insulin resistance, respectively. Rats were fasted for 6 h. For GTT, rats were given intraperitoneal injections of glucose (2 mg/kg body weight) in dH_2_O. Blood glucose levels (mg/dL) were monitored at 0, 15, 30, 60, and 120 min using a handheld blood glucose meter (FreeStyle Lite, Abbott Diabetes Care, Alameda, CA) and test strips with blood obtained *via* tail-prick. ITT was performed similarly. Rats were given intraperitoneal injections of insulin (0.35 units/kg body weight). Blood glucose levels were monitored at 0, 15, 30, and 60 min.

### 2.3. Electroretinogram (ERG)

Electroretinograms were used to measure retinal responses to light as previously described (Aung et al., 2014; Allen et al., 2015). After overnight dark adaptation and under dim red light, rats were anesthetized with ketamine (60 mg/kg) and xylazine (7.5 mg/kg), the corneal surface was anesthetized with 0.5% tetracaine, and pupils were dilated with 1% tropicamide. Platinum needle reference electrodes were placed in each cheek and a ground electrode was placed in the tail. Gold loop recording electrodes were placed on the corneas. After presentation of flash stimuli, electrical responses were recorded using a signal-averaging system (UTAS BigShot; LKC Technologies, Gaithersburg, MD). A 6-step protocol of flash stimuli presented in order of increasing luminance was used, with five dark-adapted responses recorded (scotopic: −3.0 to 2.1 log cd s/m^2^) followed by light-adaptation (30 cd/m^2^) for 10 min to saturate the rod photoreceptors. Flicker stimuli (2.0 log cd s/m^2^ at 6 Hz) were then presented in the presence of the background light to isolate cone pathway function. Rats were given atipamezole (0.5 mg/kg) following ERGs to reverse the effects of xylazine and prevent corneal ulcers (Turner and Albassam, 2005).

Right and left eye responses were averaged. Amplitudes and implicit times were measured for a-waves, b-waves, oscillatory potentials (OPs), and flicker ERG waveforms. OPs were filtered digitally (75–500 Hz band pass filter; EM Version 8.1.2, 2008; LKC Technologies) using the ERG system software and analyzed.

### 2.4. Y-maze

The Y-maze (San Diego Instruments, San Diego, CA) was used to record spontaneous alternation behavior using established methods (Maurice et al., 1995). Briefly, rats were placed in one arm of the maze and allowed to explore the entire maze freely for 8 min. All arm entries were monitored and recorded. A successful alternation was defined as entering all 3 arms consecutively. The percentage of correct spontaneous alternations was calculated: *numberofcorrectalternation* ÷ (*totalnumberofarm entries* − 2). This number represents cognitive function (short term spatial memory). The total number of entries made during an 8-min trial represents exploratory behavior.

### 2.5. Rotarod

Balance and motor coordination were assessed *via* rotarod (San Diego Instruments, San Diego, CA). Animals were given 1 min to acclimate to the stationary rotarod before testing. At the start of the test, the rod began rotating, first slowly, then gradually accelerating to 50 rpm over a 5-min period. The rotarod system software recorded the latency to fall. A trial was repeated if the rat fell within the first 15 s. At each timepoint, rats completed 4 trials per day with 5 min of rest in between. The 3 best trials were averaged for each rat.

### 2.6. Functional hyperemia

Retinal vascular function is assessed *via* functional hyperemia (Aung et al., 2012). Rats were anesthetized and their eyes prepared as for ERGs above. Rats then received an IP injection of 20 mg/kg indocyanine green (ICG, HUB Pharmaceuticals LLC, Rancho Cucamonga, CA), and their fundi were immediately imaged using the ICG filter setting on the scanning laser ophthalmoscope (Heidelberg HRA+OCT, Heidelberg, Germany). Vessel diameter was measured in response to flicker stimuli (530 nm at 12 Hz for 15 s) that induce metabolic activity of neurons. A timed series of photos was captured during flicker exposure, and the retinal vessel caliber was analyzed offline using ImageJ (US NIH, Bethesda, MD) with a Template Matching plug-in (Qingzong Tseng) and a customized MATLAB program to determine changes in response to light.

### 2.7. Vascular histology

To assess retinal vascular pathology, trypsin digest was performed (Kern et al., 2010a,b). Enucleated eyes were fixed in cold 4% paraformaldehyde overnight. The retinas were dissected carefully from eye cups and washed in distilled water overnight. Retinas were then digested in 3% crude trypsin (Difco, BD Biosciences, Franklin Lake, NJ, USA) for 2–3 h at 37°C. The tissue was placed in distilled water, and the vessels were prepared under a microscope by carefully brushing off the retinal neuronal tissue with a horse-hair brush. The vasculature was mounted on a slide and left to dry overnight. Afterward, the vessels were stained with periodic acid, Schiff–hematoxylin, and eosin, rinsed in distilled water, dehydrated in a graded alcohol series, and covered in mounting medium. Digital photographs of the retinal vessels were obtained using a light microscope. Representative images were obtained from each quadrant of the retina, and pericyte dropout (presence of pericyte ghosts) and acellular capillaries were counted.

### 2.8. HPLC

Dopamine and DOPAC levels were measured using HPLC in retinas from GK and Wistar rats euthanized at 1, 2, 8, and 12 months. Rats were euthanized between 4 and 6 h after the beginning of the light cycle to avoid diurnal differences in dopamine and DOPAC (Nir and Iuvone, 1994), and tissue was stored at −80°C. Ion-pair reverse-phase HPLC with coulometric detection was performed as described previously (Pozdeyev et al., 2008; Hendrick et al., 2020). Frozen retinas were homogenized in 0.2 N HClO4 solution containing 0.01% sodium meta-bisulfate and 25 ng/ml 3,4-dihydroxybenzylamine hydrobromide as an internal standard and centrifuged. Each supernatant fraction was separated on an Ultrasphere ODS 5 μm 250 × 4.6 mm column (Beckman Coulter, Brea, California) with a mobile phase containing 0.1 M sodium phosphate, 0.1 mM EDTA, 0.35 mM sodium octyl-sulfate, and 6% acetonitrile, pH 2.7. The dopamine and DOPAC signals from each sample were quantified using a detection curve established by standards ranging from 2 to 20 ng/ml.

### 2.9. Statistical analysis

Results are expressed as mean ± standard error of the mean (SEM). Weight, GTT, ITT, ERG, OCT, Y-maze, and rotarod results were analyzed using a two-way repeated measures (RM) ANOVA. Retinal histology, dopamine levels, DOPAC levels, and DOPAC/dopamine ratio were analyzed using a two-way ANOVA. Holms-Sidak tests were used for individual comparisons. Functional hyperemia results were analyzed using a Student’s *t*-test.

## 3. Results

### 3.1. GK rats exhibit progressive metabolic deficits beginning at 1 month

**FIGURE 1:**
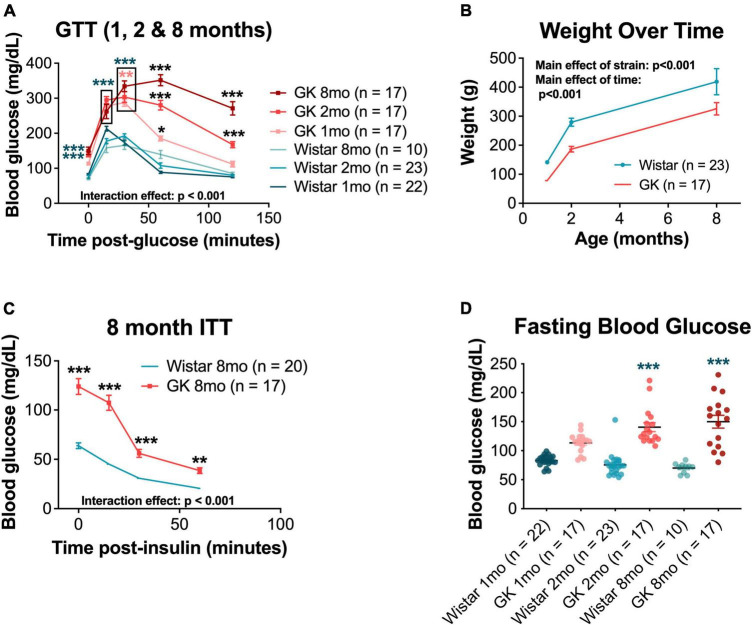
Goto-Kakizaki (GK) rats exhibit metabolic changes, including impaired glucose and insulin tolerance and reduced body weight. **(A)** Average blood glucose (mg/dL) at 0, 15, 30, 60, and 120 min post glucose injection for GTT performed at 1, 2, and 8 months of age. **(B)** Weight (g) at 1, 2, and 8 months of age. **(C)** Average blood glucose (mg/dL) at 0, 15, 30, and 60 min post insulin injection for ITT performed at 8 months of age. **(D)** Fasting blood glucose (mg/dL) at 1, 2, and 8 months of age. Black asterisks indicate comparisons between one GK group and all other groups. Blue asterisks indicate comparisons between one GK group and all Wistar groups. Pink asterisks indicate comparisons between GK groups at different ages **(A)**. *p < 0.05, **p < 0.01, ***p < 0.001. Results expressed as mean ± SEM.

Goto-Kakizaki rats showed significant impairments in glucose tolerance compared with Wistar controls, beginning at 1 month of age (*p* < 0.001) and worsening over 2 and 8 months (RM ANOVA interaction effect, strain*age, *F*_20,388_ = 17.955, *p* < 0.001; Figure 1A). GK rats did not show weight loss with hyperglycemia and continued to gain weight over their lifespan. However, GK rats weighed significantly less that Wistars at all timepoints measured (RM ANOVA main effect of strain, *F*_1,51_ = 14.401, *p* < 0.001; Figure 1B).

Additionally, GK rats showed impaired responses on ITTs compared with Wistar controls at 8 months of age (RM ANOVA interaction effect, strain*age, *F*_3,103_ = 29.214, *p* < 0.001; Figure 1C). Significant impairments in ITT were also observed at 1 and 2 months of age, but only for the 0 and 15-min timepoints (Supplementary Figure 1). GK rats also exhibited increased fasting blood glucose (the 0-min timepoint of the GTT) at 2 months (140.6 mg/dL vs. 75.5 mg/dL, *p* < 0.001) and 8 months (152.7 mg/dL vs. 70.2 mg/dL, *p* < 0.001; Figure 1D).

### 3.2. GK rats show delays in ERG implicit times

**FIGURE 2:**
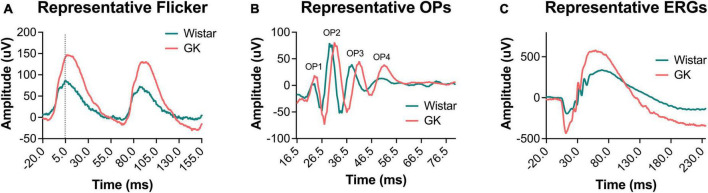
Representative waveforms from GK rats showed delayed implicit times and increased amplitudes. Representative waveforms for light-adapted flicker ERG **(A)**, dark-adapted OPs (0.7 log cd s/m^2^) **(B)**, and dark-adapted ERGs (0.7 log cd s/m^2^) **(C)** at 2 months of age.

**FIGURE 3:**
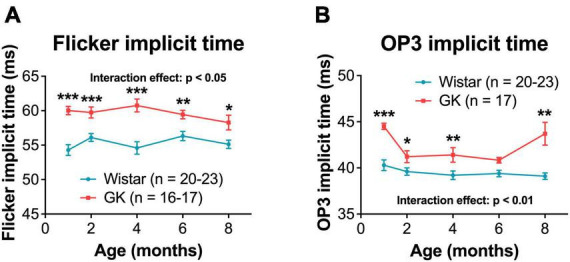
Goto-Kakizaki rats showed delayed OP and flicker implicit times, beginning at 1 month and persisting until 8 months. Quantification of flicker **(A)** and OP3 (**B**, 0.7 log cd s/m^2^) implicit times at 1, 2, 4, 6, and 8 months of age. Black asterisks indicate comparisons between GK and Wistar groups. *p < 0.05, **p < 0.01, ***p < 0.001. Results expressed as mean ± SEM.

Goto-Kakizaki rats exhibited delays in flicker and OP3 implicit times (Figures 2A, B), beginning at 1 month and persisting to 8 months (RM ANOVA interaction effect, strain*age; flicker, *F*_4,143_ = 3.307, *P* < 0.05; OP3, *F*_4,145_ = 3.437, *P* < 0.01; Figures 3A, B). Significant delays were also observed for OP2 and OP4 in GK rats (Supplementary Figure 3). Significant delays in a- and b- wave implicit time were observed at occasional time points, but these delays were not consistent from month to month (Supplementary Figure 2). While ERG results across a range of light levels were presented in our previous study, here, results for ERG and OP amplitude and implicit time are shown for the flash intensity found in our previous work to show the greatest differences between GK and Wistar rats: 0.7 log cd s/m^2^.

### 3.3. GK rats exhibit greater dark-adapted ERG amplitudes in early disease that decline over time

**FIGURE 4:**
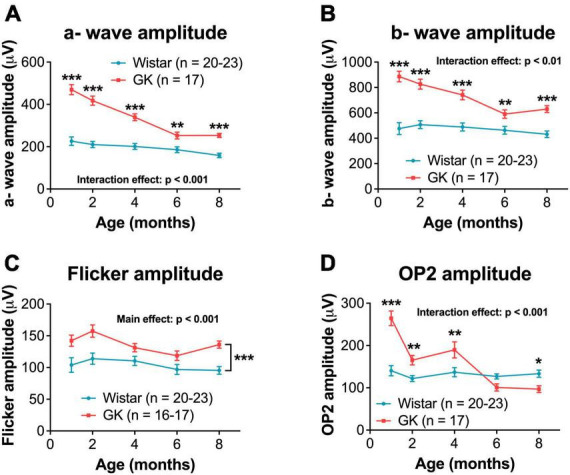
Goto-Kakizaki rats showed greater dark-adapted ERG amplitudes that declined over time. Quantification of dark-adapted a- wave **(A)**, b- wave **(B)**, flicker **(C)**, and OP2 **(D)** amplitudes at 1, 2, 4, 6, and 8 months of age. A- wave, b- wave, and OP data shown was captured at a bright flash intensity, 0.7 log cd s/m^2^. Black asterisks indicate comparisons between GK and Wistar groups. For flicker amplitude, a main effect of group was observed. Asterisks are not included at individual timepoints because the significant difference is between GK and Wistar animals across all timepoints combined. *p < 0.05, **p < 0.01, ***p < 0.001. Results expressed as mean ± SEM.

Goto-Kakizaki rats showed increased amplitudes for a- and b-waves, flicker ERG, and OPs, similar to previous reports (Allen et al., 2019; Figures 2, 4). GK a-waves and b-waves were 108 and 86% larger, respectively, at 1 month (*p* < 0.001) and were closer to control levels at 6 and 8 months (RM ANOVA interaction effect, strain*age; a-wave, *F*_4,147_ = 9.782, *p* < 0.001; b-wave, *F*_4,147_ = 4.592, *p* < 0.001; Figures 4A, B). GK rats also showed significantly larger amplitudes for flicker (RM ANOVA main effect of strain, *F*_1,143_ = 29.993, *p* < 0.001, Figure 4C) and OP1 and OP3 (Supplementary Figure 3). GK OP2 amplitudes were 88% larger at 1 month (*p* < 0.001), statistically indistinguishable at 6 months, and 27% smaller at 8 months (*p* < 0.05; RM ANOVA interaction effect, strain*age, *F*_4,145_ = 17.968, *p* < 0.001, Figure 4D).

### 3.4. GK rats exhibit cognitive and exploratory behavior deficits, but not motor function deficits

**FIGURE 5:**
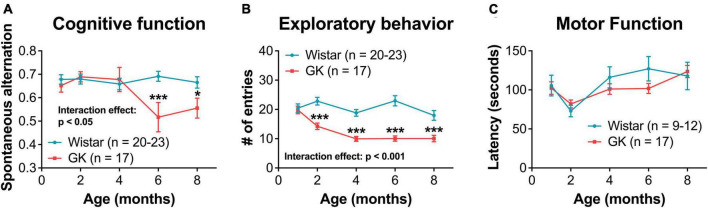
Goto-Kakizaki rats exhibit cognitive and exploratory behavior deficits, but not motor function deficits. **(A)** Cognitive function at 1, 2, 4, 6, and 8 months of age as measured by spontaneous alternation on Y-maze. **(B)** Exploratory behavior at 1, 2, 4, 6, and 8 months of age as measured by # of entries on Y-maze. **(C)** Motor coordination as measured by rotarod latency. *p < 0.05, ***p < 0.001. Results expressed as mean ± SEM.

Goto-Kakizaki rats showed significant cognitive deficits beginning at 6 months (RM ANOVA interaction effect, strain*age, *F*_4,144_ = 2.975, *P* < 0.05; Figure 5A). GK rats showed a significant reduction in exploratory behavior beginning at 2 months (RM ANOVA interaction effect, strain*age, *F*_4,144_ = 8.192, *P* < 0.001; Figure 5B). Deficits in motor coordination were not observed (Figure 5C).

### 3.5. GK rats do not show changes in vascular function by 8 months or in vascular structure by 8–12 months

**FIGURE 6:**
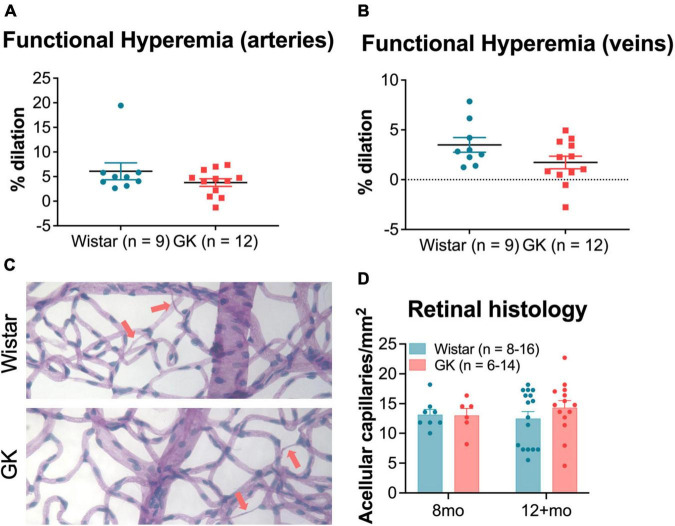
Goto-Kakizaki rats do not show changes in vascular function by 8 months or in vascular structure by 8–12 months. Functional hyperemia response (% dilation) in retinal arteries **(A)** and veins **(B)** at 8 months of age. **(C)** Representative vascular histology images from GK rats and Wistar controls. Arrows indicate acellular capillaries. **(D)** Average number of acellular capillaries/mm^2^ as counted in vascular histology images at 8 months and 12+ months. Results expressed as mean ± SEM.

In response to functional hyperemia testing, GK rats did not show a significant difference in percent dilation of retinal veins or arteries compared with Wistar rats at 8 months (Figures 6A, B). Additionally, GK rats did not show an increase in acellular capillaries in the retina at 8 or 12 months (Figures 6C, D).

### 3.6. GK rats have endogenously high levels of retinal dopamine and DOPAC

**FIGURE 7:**
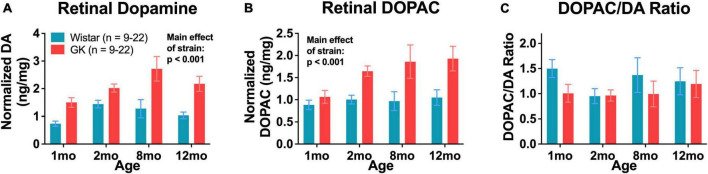
Goto-Kakizaki rats have endogenously high levels of retinal dopamine and DOPAC. Retinal dopamine **(A)** and DOPAC **(B)** levels in GK rats and Wistar controls. **(C)** DOPAC/DA ratio in GK rats and Wistar controls. Results expressed as mean ± SEM.

Retinal dopamine levels in GK rats were significantly higher than age-matched Wistar controls as measured by HPLC at 1, 2, 8, and 12 months of age (RM ANOVA main effect of strain, *F*_1,108_ = 45.152, *P* < 0.001; Figure 7A). Retinal DOPAC levels in GK rats were significantly higher as well, though this change appeared to begin at 2 months (RM ANOVA main effect of strain, *F*_1,108_ = 26.611, *P* < 0.001; Figure 7B). The DOPAC/dopamine ratio did not differ between GK and Wistar rats (Figure 7C).

## 4. Discussion

This study confirms our previous work by demonstrating that retinal neuronal function changes precede cognitive changes in the GK rat. Novel findings include the long-term characterization of retinal and cognitive deficits, lack of vascular alterations, and elevated dopamine levels in GK rats.

### 4.1. Retinal function changes occurred before retinal vascular and cognitive changes in the GK rat model of type II diabetes

After the onset of hyperglycemia, retinal function deficits appeared first in GK rats, with significant delays in flicker and OP implicit times as early as 1 month of age. Reduced exploratory behavior and spatial cognition were observed at 2 and 6 months, respectively. Changes in motor function were not observed. This work confirms our previous study, which showed that retinal function deficits presented before cognitive deficits in GK rats followed until 8 weeks of age (Allen et al., 2019). In the current study, we followed GK rats out to 8 months and 12+ months in order to assess retinal vascular pathology.

Retinal function changes were shown to precede changes in retinal vascular function and histology in GK rats. Previous research from our group and others has shown that retinal neuronal dysfunction occurs prior to retinal vascular pathology in diabetes, both in rodent models (Feng et al., 2009; Aung et al., 2013; Pardue et al., 2014; Chesler et al., 2021) and in patients (Pardue et al., 2014; Gella et al., 2015; Wolff et al., 2015; Stem et al., 2016; Barber and Baccouche, 2017).

A discrepancy between this study and our previous work is the timing of the appearance of cognitive deficits in the GK rat. We previously showed that the GK rat exhibits cognitive deficits beginning at 7 weeks of age (Allen et al., 2019). Meanwhile, here, cognitive deficits were not exhibited until 6 months of age. In the previous study, Y-maze testing was performed weekly, and here, Y-maze testing was performed monthly. Based on this comparison, as well as other work from our group, the Y-maze is much more sensitive to changes between experimental groups when used on a weekly basis vs. a monthly basis. We suspect this is due to animal familiarity with the test. The control rats in our first paper show a slight increase in cognitive scores from week 4 to week 8 that is not observed with the monthly testing performed here.

While the delays in flicker and OP implicit times observed here are typical of diabetic retinopathy (Aung et al., 2013; Pardue et al., 2014; Motz et al., 2020; Chesler et al., 2021), the supernormal ERG amplitudes we observed in GK rats are unusual and confirm our previous work in this model (Allen et al., 2019). Small increases in ERG amplitude have sometimes been reported in patients with early DR (Jenkins and Cartwright, 1990), but ERG amplitudes that are twice that of controls are unusual and a potential limitation of using the GK model. As we previously speculated (Allen et al., 2019), because the GK rat exhibits metabolic changes as early as 2 weeks, the hyperglycemia may affect retinal development and circuitry in a way that increases ERG amplitudes, perhaps by targeting inhibitory pathways. Additionally, the increased a- and b- wave amplitudes could be caused by hyperglycemia inducing changes in distinct outer retinal mechanisms. For example, perhaps the photoreceptors developed to be more active because there was more energy (glucose) around during development. Photoreceptors are metabolically needy cells, and perhaps the additional glucose resulted in changes at the synapse or channel level, leading to amplified responses.

Interestingly, the increases in amplitude appear to follow a similar time course, with amplitude differences in GK rats being largest at 1 and 2 months and closer to Wistar levels at 6 and 8 months for a- waves, b- waves, OP1, and OP2. Often, the amplitudes for the two groups are closest at 6 months, with OP2 even dropping below control levels for GK rats at 8 months. Further research is needed to determine the specific changes in retinal circuitry that are driving these ERG results.

A limitation of using GK and Wistar rats is that ERGs are performed based on age. This means that GK and Wistar ERGs are not performed on the same day, which could cause additional variability in ERG data. We address this issue by using a large n that includes multiple litters. Additionally, we report findings that are observed consistently across multiple timepoints (Figures 3, 4). Findings without a consistent pattern (i.e., a- and b- wave implicit time, Supplementary Figure 2) are shown for sake of completion but are not emphasized. This variability may also play into findings like the oscillatory potential (OP3) implicit time measurements (Figure 3B), as we are unsure why the greatest differences in implicit time would occur at 1 and 8 months. Instead, we view this data as a whole and note the delay in implicit time begins at 1 month and persists over time.

This same limitation can introduce more variability into our cognitive assay as well and may explain why the impairment in cognitive function is less pronounced at 8 months than 6 months. Additionally, the cognitive portion of the Y-maze task is a more variable test, in general, as evidenced by the larger error bars, which are overlapping for the 6- and 8-month GK timepoints.

If retinal dysfunction appears prior to other functional complications in diabetes, treatments could be implemented at the first sign of retinal deficits to slow or prevent cognitive deficits and retinal vascular pathology (Motz et al., 2020). A similar window could be identified in people with diabetes, allowing for earlier treatment of complications.

### 4.2. GK rats did not show retinal vascular pathology typical of diabetes

In agreement with previous research (Oshitari et al., 2009; van Dijk et al., 2010; Aung et al., 2013; Pardue et al., 2014; Barber and Baccouche, 2017), we identified retinal function deficits prior to the development of retinal vascular pathology. Unlike previous research, we did not observe retinal vascular pathology in the GK rats, even at later stages of disease. The functional hyperemia response measures dilation of retinal veins and arteries in response to flickering green light and is thought to be mediated by neurovascular coupling. GK rats did not exhibit changes in functional hyperemia in retinal veins or arteries at 8 months of age. Meanwhile, abnormal functional hyperemia is observed after two weeks (Aung et al., 2012) of diabetes in STZ Type I mice and 7 months of diabetes in STZ Type I rats (Mishra and Newman, 2010, 2012). A reduced functional hyperemia response has also been shown to occur in patients prior to clinical retinopathy (Garhöfer et al., 2004; Pemp et al., 2009).

Similarly, GK rats did not show retinal vascular pathology as measured by numbers of acellular capillaries at 8 months or 12+ months. In contrast, STZ Type I rats show increased numbers of acellular capillaries in the retina at 8 months post-diabetes for Wistar and Lewis strains (Kern et al., 2010a) and at least 12 months post-diabetes for Sprague-Dawley rats (Kowluru et al., 2001). Increased numbers of acellular capillaries have been reported in Type II diabetic rodents as well, including Zucker diabetic fatty rats at 8 months (Wohlfart et al., 2014) and *db/db* mice at 11 months (Beli et al., 2018). While rodent models of diabetes do not show all of the retinal vascular changes observed in patients with diabetes (hemorrhages, neovascularization, etc.), acellular capillaries and pericyte dropout are typically observed and are thought to be the rodent equivalent of clinical vascular pathology in patients. The absence of this retinal vascular pathology in animals with confirmed hyperglycemia lasting 12+ months is extremely unusual, particularly in light of the changes in retinal and cognitive function.

### 4.3. GK rats exhibit endogenously high levels of retinal dopamine and DOPAC

Goto-Kakizaki rats showed higher than normal levels of retinal dopamine, twice as high as Wistar controls on average. GK rats also showed increased retinal levels of DOPAC, a metabolite of dopamine. However, the DOPAC/dopamine ratios were the same in GK and Wistar rats, indicating that while overall dopamine and DOPAC levels are higher, dopamine turnover may not be impaired. In contrast, other models of diabetes are reported to have a reduction in retinal dopamine. STZ Type I rats exhibit decreased levels of dopamine and its metabolites (Aung et al., 2014), and a loss of dopaminergic amacrine cells (Gastinger et al., 2006). Dopamine deficiency in the retina has been reported in STZ mice (Kim et al., 2018) and Ins2Akita mice as well (Gastinger et al., 2006). Indeed, dopamine deficiency appears to underlie early retinal function deficits in diabetes as treating diabetic animals with L-DOPA delays retinal dysfunction (Aung et al., 2014). It is possible that the lack of retinal vascular pathology in GK rats and the endogenously high levels of retinal dopamine are connected. However, even with elevated dopamine levels, retinal neuronal function and cognitive function were impaired in this model. Treatments that target dopamine could be used in an alternate model of Type II diabetes to better assess the effects of dopamine on the degree of impairment in retinal and cognitive function.

### 4.4. Dopamine as an anti-angiogenic in other disease models

Dopamine and/or dopamine agonists have been shown to act as potent anti-angiogenics in models of breast, colon (Sarkar et al., 2015), and lung cancer (Hoeppner et al., 2015), melanoma (Basu et al., 2004), pulmonary edema (Vohra et al., 2012), ulcerative colitis (Tolstanova et al., 2015), and ovarian hyperstimulation syndrome (Ferrero et al., 2014). Dopamine has been shown to reduce vascular permeability in endothelial cells (Bhattacharya et al., 2008) and attenuate vascular leakage by inhibiting VEGF, a potent stimulator of angiogenesis, at the post-transcriptional level (Ferrero et al., 2014). Specifically, dopamine treatment inhibits VEGF secretion (Ferrero et al., 2014) and prevents VEGF binding to VEGF receptor 2 by dephosphorylating the receptor (Sinha et al., 2009) and stimulating its endocytosis (Basu et al., 2001).

We were only able to find two studies that point to dopamine as an anti-angiogenic in the retina: a retrospective study showing that age-related macular degeneration was delayed by approximately 8 years in patients who were taking L-DOPA for Parkinson’s disease (Brilliant et al., 2016) and an animal study showing that the dopamine agonist bromocriptine reduces vascular permeability in diabetic mice (Kern et al., 2021). The research presented here suggests that treatments that target dopamine could be used as in the diabetic retina to reduce or prevent neovascularization and other vascular pathology, the clinical hallmark of DR and a cause of blindness.

### 4.5. Neuronal and vascular changes may not be related in the GK rat

Clinically, diabetes is viewed as a vascular disorder (Frank, 2004; Tonade and Kern, 2021). However, more recent evidence illustrates neuronal changes in diabetic retinopathy (Holopigian et al., 1992, 1997; Ghirlanda et al., 1997; Shirao and Kawasaki, 1998; Barber, 2003; Antonetti et al., 2006; Lecleire-Collet et al., 2011; Aung et al., 2013; Pardue et al., 2014). Additionally, neurovascular coupling is thought to be critical to proper retinal function and is impaired with diabetic retinopathy (Riva et al., 2005). The research presented here is a rare instance of a diabetes model where neuronal and vascular changes are not linked.

### 4.6. Limitations of the GK rat as a model of type II diabetes

The GK rat exhibits many features that make it an attractive model for Type II diabetes, including the fact that it is non-obese, non-insulin dependent, polygenic, and spontaneously develops impaired insulin secretion by 2 weeks with impaired fasting hyperglycemia by 4 weeks. The GK rat has also been shown to display retinal (Matsubara et al., 2006; Allen et al., 2019), cognitive (Moreira et al., 2007; Li et al., 2013; Allen et al., 2019), and motor nerve deficits (Suzuki et al., 1990).

However, our results showed that GK rats lack critical features of diabetic retinopathy, including characteristic vascular pathology, loss of dopamine, linked neuronal and vascular changes, and possible developmental alterations leading to unusually high ERG amplitudes. While still useful for modeling other aspects of diabetes, this study suggests that other models of Type II diabetes should be considered when investigating diabetic retinopathy. Future investigation of dopamine treatments for diabetic retinopathy will focus on a Type II model of diabetes that exhibits deficits that more closely model diabetic retinopathy.

## 5. Conclusion

As shown previously, retinal function deficits appeared prior to cognitive deficits and vascular pathology in GK rats. Despite showing defects in retinal neuronal function, GK rats did not show changes in retinal vascular function or vascular histology. Higher endogenous levels of retinal dopamine in GK rats may be conferring protection against vascular pathology, and GK rats could be treated with a dopamine antagonist and assessed for vascular changes to confirm a causal relationship. Future research will investigate the potential of dopamine as an anti-angiogenic in the diabetic retina.

## Data availability statement

The raw data supporting the conclusions of this article will be made available by the authors, without undue reservation.

## Ethics statement

The animal study was reviewed and approved by the Atlanta Veterans Affairs Institutional Animal Care and Use Committee.

## Author contributions

RA, MP, PT, and PI designed the research. RA, CK, and AF performed the animal assessments. AW performed the histology. RA and AG wrote the manuscript. RA, CK, KC, AW, and AG performed the data analysis. LH, JD, and PI provided the HPLC. TK provided the vascular histology. RA and MP performed the statistical analysis. MP supervised the experiments and the production of the figures and the manuscript. All authors contributed to the article and approved the submitted version.
