# Supplementary material for: Diabetic rats with high levels of endogenous dopamine do not show retinal vascular pathology
Source: Front Neurosci. 2023 Mar 22;17:1125784. doi: 10.3389/fnins.2023.1125784 (PMC10073440; doi:10.3389/fnins.2023.1125784)
Supplement: Supplementary file 1 [file Data_Sheet_1.docx]

Title: Diabetic rats with high levels of endogenous dopamine do not show retinal vascular pathology

Short title: Dopamine and diabetic retinopathy

Authors:

*Rachael S Allen, PhD^a,b^, Cara T Motz, BS^a^, Andrew J Feola, PhD^a,b,f^, Alice S Win^a,b^, BS, Allison R Grubman^a,f^, Kyle C Chesler^a,b^, BS, Li He^f,g^, PhD, Jendayi A Dixon^f,g^, BS, Timothy S Kern^c,d,e^, PhD, P Michael Iuvone^f,g^, PhD, Peter M Thule, MD^h^, Machelle T Pardue, PhD^a,b,f^


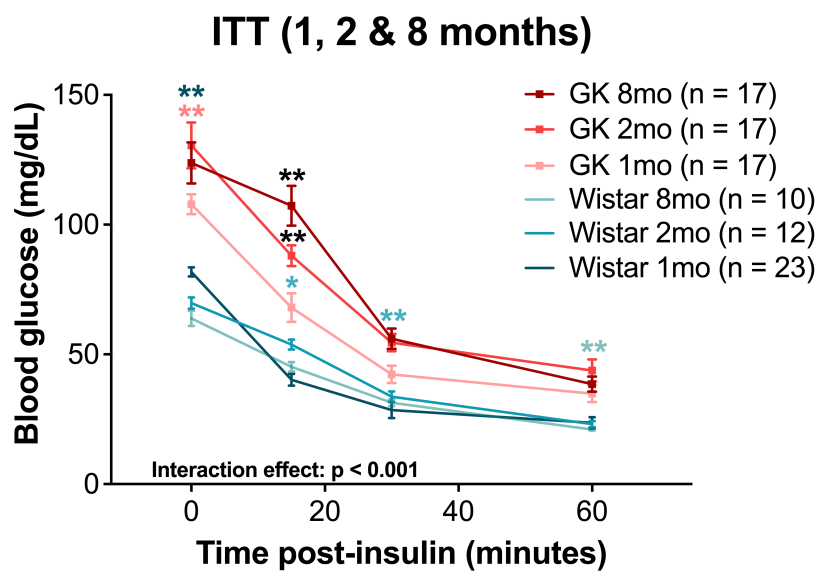


**Supplemental Figure S1. GK rats exhibit impaired insulin tolerance.** Average blood glucose (mg/dL) at 0, 15, 30, and 60 minutes post insulin injection for ITT performed at 1, 2, and 8 months of age.

At 0 minutes: All GK are different from all Wistar groups. 2 & 8-month GK are different from 1-month GK.

At 15 minutes: 2 & 8-month GK are different from all other groups. 1-month GK are different from 1 & 8-month Wistar groups.

At 30 minutes: 2 & 8-month GK are different from 1 & 8-month Wistar groups.

At 60 minutes: 2-month GK are different from the 8-month Wistar group.

Black asterisks indicate comparisons between one GK group and all other groups. Blue asterisks indicate comparisons between one GK group and Wistar groups. Pink asterisks indicate comparisons between GK groups at different ages. * p < 0.05, ** p < 0.01, *** p < 0.001. Results expressed as mean ± SEM.


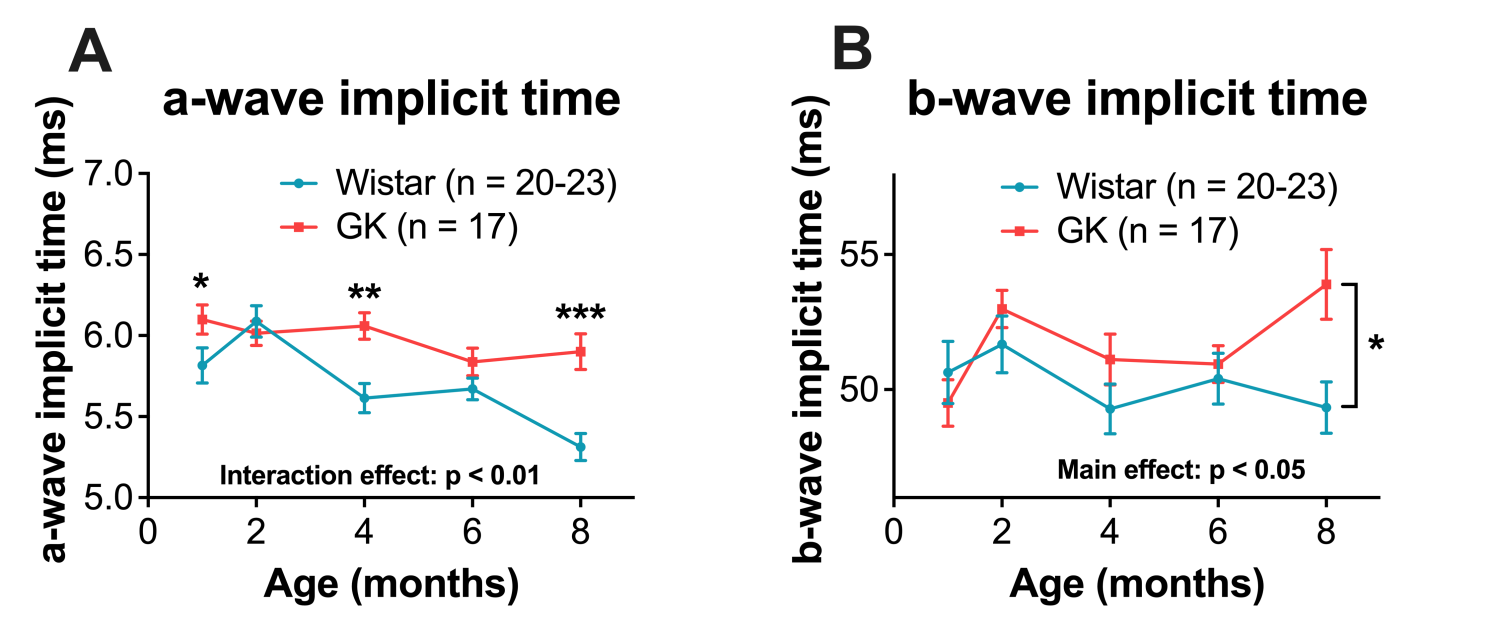


**Supplemental Figure S2. ERG implicit times for a- and b- waves for GK and Wistar rats over time.** Quantification of a- wave (A) and b- wave (B) implicit times at 1, 2, 4, 6, and 8 months of age at 0.7 log cd s/m^2^. Black asterisks indicate comparisons between GK and Wistar groups. For b- wave implicit time, a main effect of group was observed. Asterisks are not included at individual timepoints because the significant difference is between GK and Wistar animals across all timepoints combined. * p < 0.05, ** p < 0.01, *** p < 0.001. Results expressed as mean ± SEM.


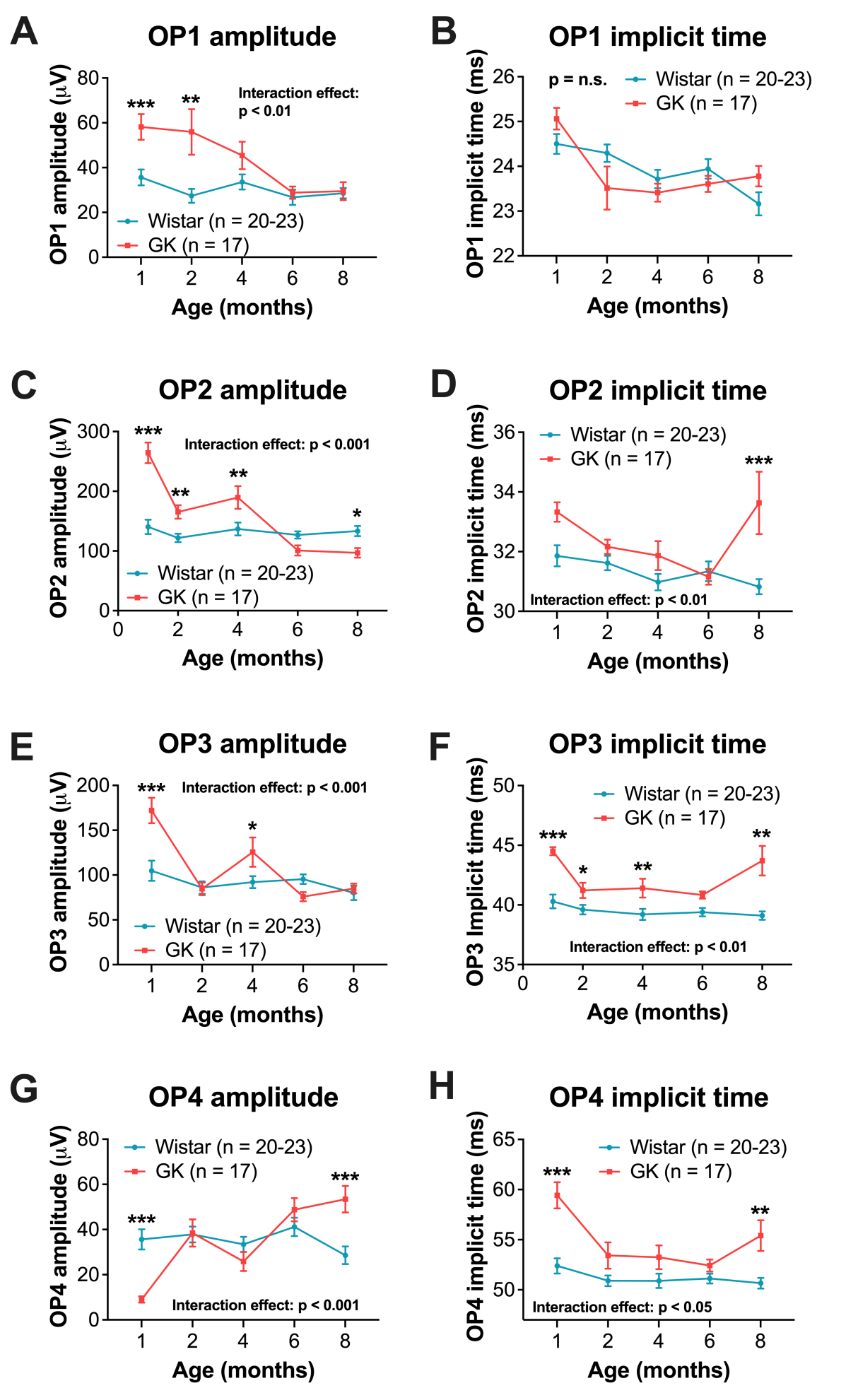


**Supplemental Figure S3. ERG oscillatory potential amplitudes and implicit times for GK and Wistar rats over time.** Quantification of amplitudes (A, C, E, G) and implicit times (B, D, F, H) for OP1 (A, B), OP2 (C, D), OP3 (E, F), and OP4 (G, H) at 1, 2, 4, 6, and 8 months of age at 0.7 log cd s/m^2^. Black asterisks indicate comparisons between GK and Wistar groups. * p < 0.05, ** p < 0.01, *** p < 0.001. Results expressed as mean ± SEM.
